# Synchronous Bilateral Shamblin Type III Carotid Body Tumors and Adrenal Pheochromocytoma with SDHD Mutation: A Rare Presentation, with Multimodality Imaging Findings

**DOI:** 10.1055/s-0045-1814731

**Published:** 2026-01-05

**Authors:** Roopal Agrawal, Keerti Sitani, Sandip Basu

**Affiliations:** 1 Radiation Medicine Centre, Bhabha Atomic Research Centre, Tata Memorial Centre Annexe, Parel, Mumbai, Maharashtra, India; 2 Homi Bhabha National Institute, Mumbai, Maharashtra, India

**Keywords:** carotid body tumors, PET/CT, ^18^ F-FDG, ^68^ Ga-DOTATATE, ^131^ I-MIBG, pheochromocytoma, Ki-67, histopathological analysis

## Abstract

Pheochromocytomas and paragangliomas are relatively rare tumors, with an incidence of approximately 0.6 cases per 100,000 person-years. Moreover, the co-occurrence of these tumors is extremely rare and is often associated with pheochromocytomas/paragangliomas-related pathogenic mutations. We present the case of a 33-year-old female diagnosed with bilateral carotid body paragangliomas and a concurrently detected pheochromocytoma, with metastases to the abdominal lymph nodes and lung. DNA analysis revealed a mutation in the succinate dehydrogenase subunit D gene. The tumors displayed high-grade SSTR expression (Krenning grade 4 uptake) on Gallium-68 [⁶⁸Ga]-DOTA-(Tyr ^3^ )-octreotate Positron Emission Tomography/Computed Tomography, with no significant tracer concentration on the I-131 MIBG scan. Another notable feature in this case was the visually evident intra- and inter-tumoral metabolic heterogeneity on ^18^ F-fluorodeoxyglucose positron emission tomography/computed tomography, especially within the multiple carotid paragangliomas. With the adoption of a multimodality diagnostic approach (MRI, FDG PET/CT, SSTR PET/CT, and I-131 MIBG scintigraphy), a holistic theranostic approach was employed with the most rational therapeutic option offered to the patient.

## Introduction

Paragangliomas and pheochromocytomas are neuroendocrine-origin neoplasms that may arise sporadically or in association with hereditary syndromes. Paragangliomas are either derived from the paravertebral sympathetic chains or parasympathetic ganglia in the body, whereas pheochromocytomas arise from chromaffin cells of the adrenal medulla. 1 2

Clinical manifestations may vary depending on the location and functionality of these tumors. Sympathetic paragangliomas and pheochromocytomas typically present with symptoms attributable to catecholamine excess, such as blood pressure fluctuations (episodic hypertension or orthostatic hypotension), palpitations, diaphoresis, and headache. 3 On the other hand, head and neck paragangliomas are usually non-functioning and more commonly discovered incidentally during imaging studies. When symptomatic, they present with compressive or infiltrative symptoms on adjacent structures, causing hearing loss, tinnitus, dysphagia, and even cranial nerve palsies. 1

Anatomical and functional imaging play complementary roles in the diagnosis, staging, and treatment planning of pheochromocytomas/paragangliomas (PPGLs). Functional radionuclide imaging offers a range of radiotracers, such as Gallium-68 [ ^68^ Ga]-DOTA-(Tyr ^3^ )-octreotate Positron Emission Tomography/Computed Tomography ( ^68^ Ga-DOTATATE), ^131^ I-metaiodobenzylguanidine ( ^123^ I-MIBG), ^18^ F-fluorodeoxyglucose positron emission tomography/computed tomography ( ^18^ F-FDG PET/CT), and ^18^ F-Flourodopamine (18F-FDOPA), tailored to imagine the underlying molecular characteristics and pathogenic pathways of these neoplasms. 4

Herein, we present a case of bilateral carotid body paragangliomas with synchronous retroperitoneal pheochromocytoma in a young female, associated with a succinate dehydrogenase subunit D (SDHD) mutation. The presentation is relatively rare, with only a few cases reported worldwide. 5 This report aims to highlight the importance of multimodality functional imaging and genetic analysis for the characterization and optimization of treatment strategies in such complex paraganglioma–pheochromocytoma syndromes.

## Case Report

A 33-year-old-female presented with bilateral neck swellings that has been present for the past 3 years, with a rapid increase in size over the previous 3 to 4 weeks. The patient also reported a history of multiple episodes of postural hypotension and a single episode of accelerated hypertension. On biochemical evaluation, plasma normetanephrine levels were elevated (760 pg/mL).

Magnetic resonance imaging (MRI) of the neck revealed two well-defined lobulated soft-tissue masses located at the bilateral carotid bifurcations, measuring approximately 3.9 × 4 × 8.6 cm on the left side and 2.5 × 2.7 × 4.1 cm on the right side (AP × TR × CC). Characteristic splaying of the internal and external carotid arteries, with 100% vascular encasement, was noted bilaterally. A diagnosis of bilateral carotid paragangliomas (100% encasement of carotid arteries) was made based on clinical and imaging findings. Biopsy for histopathological analysis was intentionally deferred, considering the high risk of intraoperative bleeding and potential catecholamine crisis associated with functional paragangliomas, particularly in those with extensive vascular encasement.

**Fig. 1:**
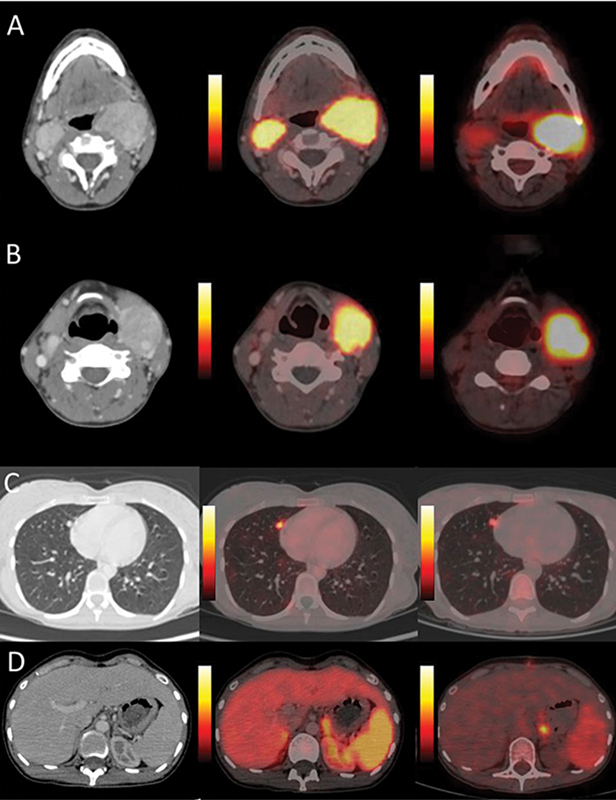
Dual-tracer transaxial PET/CT images showing SSTR-expressing and ^18^ F-FDG–avid bilateral carotid body paragangliomas ( **A** and **B** ); similar 18F-FDG-concentrating and SSTR-expressing parenchymal nodule located in the middle lobe of the right lung ( **C** ); and a left-sided para-aortic lymph node ( **D** ). ^18^ F-FDG, ^18^ F-fluorodeoxyglucose; PET/CT, positron emission tomography/computed tomography; SSTR, somatostatin receptors.

Diagnostic ^68^ Ga-DOTATATE PET/CT was performed for disease characterization and staging, which revealed intense SSTR expression (Krenning grade 4) in the bilateral carotid paragangliomas. Additionally, an SSTR expressing soft-tissue lesion was noted in the retroperitoneal aortocaval region, measuring approximately 2 × 1.6 cm, without any evidence of locoregional invasion of adjacent structures. A few SSTR expressing locoregional retroperitoneal lymph nodes were also noted. Moreover, multiple SSTR-expressing soft-tissue nodules were scattered in the bilateral lung parenchyma ( Fig. 1 ).

**Fig. 2:**
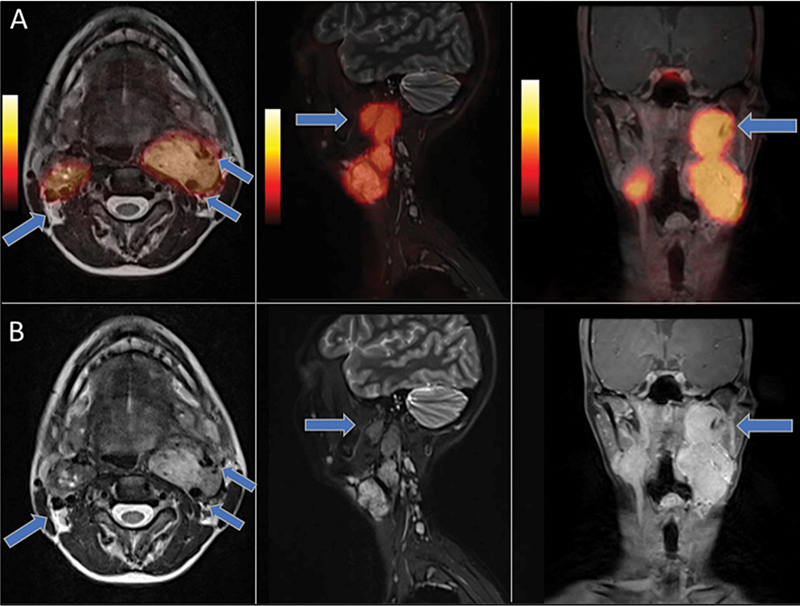
Fusion axial, sagittal, and coronal ^68^ Ga-DOTATATE PET/MR ( **A** ) and T2 sequence MR ( **B** ) image sections demonstrate SSTR expressing bilateral lobular soft-tissue masses causing complete encasement of the bilateral internal and external carotid arteries ( *blue arrows* ), classified as Shamblin type III tumors. ^68^ Ga-DOTATATE PET/CT, Gallium-68 [ ^68^ Ga]-DOTA-(Tyr ^3^ )-octreotate Positron Emission Tomography/Computed Tomography; SSTR, somatostatin receptors.

**Fig. 3:**
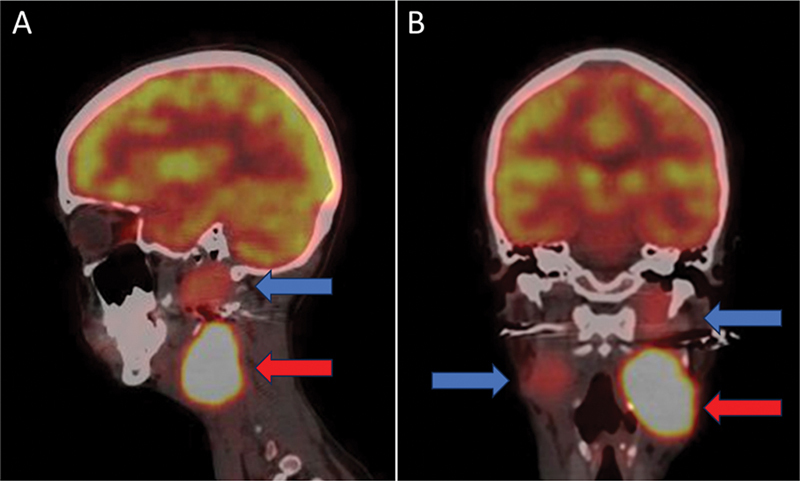
Sagittal ( **A** ) and coronal ( **B** ) fusion ^18^ F-FDG PET/CT cross section images demonstrate low-grade tracer uptake in the right-sided and upper half of the left carotid paraganglioma ( *blue arrows* ) with high-grade metabolic uptake in the lower half of the left-sided tumor ( *red arrows* ), demonstrating significant inter- and intra-tumoral heterogeneity. ^18^ F-FDG PET/CT, ^18^ F-fluorodeoxyglucose positron emission tomography/computed tomography.

Excision of the retroperitoneal mass was done at a different center, and histopathological analysis was suggestive of pheochromocytoma, with a Ki-67 index of 2 to 3%. Genetic sequencing revealed a mutation in the succinate *SDHD* gene. Considering the extensive encasement of the bilateral carotid arteries leading to inoperability of the tumors, and extensive metastatic disease, the patient was referred for systemic therapy. ^18^ F-FDG PET/CT scan was performed to further assess tumor biology, revealing heterogenous low-to-moderate grade tracer uptake in the bilateral carotid paragangliomas, left para-aortic lymph node, and few lung nodules ( Fig. 2 ). ^131^ I-MIBG scintigraphy performed for theranostic evaluation, revealed no significant radiotracer uptake in the bilateral carotid body tumors or metastatic lesions ( Fig. 3 ).

## Discussion

The diagnostic evaluation of PPGLs involves a multidisciplinary approach, including biochemical markers, anatomical and functional imaging, and genetic analysis. Biochemical assessment includes measurement of plasma-free metanephrines and normetanephrines, or urinary fractionated metanephrines, and is primarily used for screening purposes. Anatomical imaging using MRI or CT aids in the localization and delineation of the primary tumors, with MRI being preferred for head and neck paragangliomas due to its superior soft-tissue resolution and lack of radiation exposure. 6

Functional imaging is essential for disease characterization, detection of small metastatic foci, and prognostication. ^68^ Ga-DOTA-conjugated somatostatin analogues imaging has proven to be highly sensitive for the detection of sporadic PPGLs, especially in cases with SDHx-associated mutations. 7 This modality is particularly useful for the evaluation of lesions where histopathological analysis is not feasible, such as the bilateral carotid body tumors and multiple lung nodules in this patient.

On the other hand, ^18^ F-FDG PET/CT is valuable for the assessment of metastatic or aggressive disease, especially in succinate dehydrogenase (SDH) subtype-B mutation carriers. ^18^ F-FDOPA PET/CT imaging offers excellent sensitivity for imaging both hereditary and sporadic pheochromocytomas, in addition to imaging head and neck paragangliomas. 8 Although ^123^ I-MIBG imaging holds high specificity for imaging adrenal pheochromocytomas, and has theranostic implications in patient selection for targeted ^131^ I-MIBG therapy, its limited sensitivity may lead to underestimation of disease burden in many cases. 9

Approximately 40% of PPGLs have a hereditary basis, involving multiple germline mutations and syndromic associations such as von Hippel–Lindau, multiple endocrine neoplasia type 2 (RET), neurofibromatosis type 1, and hereditary paraganglioma–pheochromocytoma syndrome. 10 Notably, pathogenic mutations in the SDH complex subunits (SDHA, SDHB, SDHC, SDHD) are strongly associated with aggressive hereditary PPGL syndromes. SDHD mutations exhibit a strong predilection for head and neck paragangliomas, with a higher risk of metastatic potential compared to sporadic cases. 11 These findings were in concordance with the histopathological findings in our case.

**Fig. 4:**
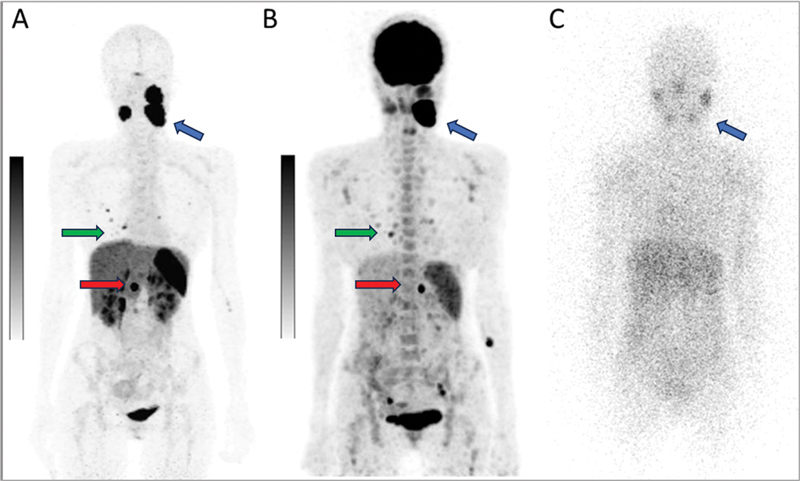
Maximum-intensity projection images of pre-operative ^68^ Ga-DOTATATE PET/CT ( **A** ) show intense SSTR expression in bilateral carotid paragangliomas ( *blue arrow* ), pheochromocytoma ( *red arrow* ), and few lung nodules ( *green arrow* ); postoperative ^18^ F-FDG PET/CT ( **B** ) shows heterogenous moderate- to high-grade metabolic uptake in the abovementioned lesions with an anterior planar ^131^ I-MIBG scan ( **C** ) image demonstrating faint tracer concentration in the bilateral carotid paragangliomas ( *blue arrow* ). ^18^ F-FDG PET/CT, ^18^ F-fluorodeoxyglucose positron emission tomography/computed tomography; ^68^ Ga-DOTATATE PET/CT, Gallium-68 [ ^68^ Ga]-DOTA-(Tyr ^3^ )-octreotate Positron Emission Tomography/Computed Tomography; SSTR, somatostatin receptors.

The management of bilateral carotid body tumors or multifocal PPGLs has not yet been standardized. The current mainstay of treatment for carotid body tumors involves staged excision, with adequate preoperative alpha- and beta-adrenergic blockade, 12 although this option is associated with a significant risk of hemorrhage and neurovascular complications (especially in Shamblin type III tumors). 13 Other therapeutic options for locally advanced, multifocal, or metastatic disease include observation, intensity-modulated radiotherapy, radionuclide therapy—including peptide receptor radionuclide therapy (PRRT) or MIBG therapy—chemotherapy, and/or novel targeted therapies (e.g., tyrosine kinase inhibitors). 14 15 In our case, considering the intense SSTR expression and absence of MIBG avidity ( Fig. 3 ), the patient was deemed unsuitable for MIBG-based therapy. She was therefore planned for PRRT with ^177^ Lu-DOTATATE ( Fig. 4 ). These findings highlight the importance of using complementary imaging modalities for accurate staging and treatment planning in PPGLs.

## Conclusion

In summary, this case illustrates a rare presentation of SDHD-mutated hereditary paraganglioma–pheochromocytoma syndrome, presenting as bilateral Shamblin III carotid body paragangliomas, with synchronous retroperitoneal pheochromocytoma and distant metastatic lesions in a young female. This case report highlights the importance of comprehensive multimodality functional imaging to understand disease pathology, evaluate inter- and intra-tumoral heterogeneity, and subsequently select the best therapeutic options based on the degree of uptake in metastatic lesions. Malignancies such as neuroendocrine tumors and paragangliomas may display variable patterns of tracer uptake, guiding personalized treatment choices for different patients, facilitated by nuclear medicine and molecular imaging.
